# Three nested randomized controlled trials of peer-only or multiple stakeholder group feedback within Delphi surveys during core outcome and information set development

**DOI:** 10.1186/s13063-016-1479-x

**Published:** 2016-08-17

**Authors:** Sara T. Brookes, Rhiannon C. Macefield, Paula R. Williamson, Angus G. McNair, Shelley Potter, Natalie S. Blencowe, Sean Strong, Jane M. Blazeby

**Affiliations:** 1 School of Social and Community Medicine, University of Bristol, Canynge Hall, 39 Whatley Road, Bristol, BS8 2PS UK; 2 MRC North West Hub for Trials Methodology Research, Department of Biostatistics, University of Liverpool, 1st floor Duncan Building, Daulby Street, Liverpool, L69 3GA UK; 3 Division of Surgery, Head and Neck, University Hospitals Bristol NHS Foundation Trust, Upper Mauldin Street, Bristol, BS2 8HW UK

**Keywords:** Core outcome set, Core information set, Delphi, Consensus, Feedback

## Abstract

**Background:**

Methods for developing a core outcome or information set require involvement of key stakeholders to prioritise many items and achieve agreement as to the core set. The Delphi technique requires participants to rate the importance of items in sequential questionnaires (or rounds) with feedback provided in each subsequent round such that participants are able to consider the views of others. This study examines the impact of receiving feedback from different stakeholder groups, on the subsequent rating of items and the level of agreement between stakeholders.

**Methods:**

Randomized controlled trials were nested within the development of three core sets each including a Delphi process with two rounds of questionnaires, completed by patients and health professionals. Participants rated items from 1 (not essential) to 9 (absolutely essential). For round 2, participants were randomized to receive feedback from their peer stakeholder group only (peer) or both stakeholder groups separately (multiple). Decisions as to which items to retain following each round were determined by pre-specified criteria.

**Results:**

Whilst type of feedback did not impact on the percentage of items for which a participant subsequently changed their rating, or the magnitude of change, it did impact on items retained at the end of round 2. Each core set contained discordant items retained by one feedback group but not the other (3–22 % discordant items). Consensus between patients and professionals in items to retain was greater amongst those receiving multiple group feedback in each core set (65–82 % agreement for peer-only feedback versus 74–94 % for multiple feedback). In addition, differences in round 2 scores were smaller between stakeholder groups receiving multiple feedback than between those receiving peer group feedback only. Variability in item scores across stakeholders was reduced following any feedback but this reduction was consistently greater amongst the multiple feedback group.

**Conclusions:**

In the development of a core outcome or information set, providing feedback within Delphi questionnaires from all stakeholder groups separately may influence the final core set and improve consensus between the groups. Further work is needed to better understand how participants rate and re-rate items within a Delphi process.

**Trial registration:**

The three randomized controlled trials reported here were each nested within the development of a core information or outcome set to investigate processes in core outcome and information set development. Outcomes were not health-related and therefore trial registration was not applicable.

**Electronic supplementary material:**

The online version of this article (doi:10.1186/s13063-016-1479-x) contains supplementary material, which is available to authorized users.

## Background

A core outcome set (COS) is an agreed minimum set of outcomes to be measured and reported in all clinical effectiveness trials of a particular condition or intervention [1]. A COS requires incorporation of patient opinion as well as that of health professionals to ensure that outcomes are patient centred and relevant to healthcare. The widespread implementation of such sets will reduce heterogeneity in reported outcomes and better enable data synthesis. Methods to develop COSs have been promoted by the COMET initiative [1, 2] and have also been used for the development of core information sets (CISs) (a minimum set of information to use in all consultations as a baseline for information provision for treatment [3–5]). The methods for developing CISs and COSs are very similar and both involve working with key stakeholders to prioritise large numbers of items (outcomes or information) and achieve agreement as to the core set.

A recent review of studies developing core outcome sets for use in clinical trials, found that 31 % incorporated a Delphi process [6]. The Delphi technique [7] (when used within questionnaire surveys) requires participants to anonymously rate the importance of different items in sequential questionnaires or ‘rounds’ sent by post or electronically. After each round, responses for each item are summarized and fed back (anonymously) within the subsequent questionnaire (the next round), enabling participants to consider the views of others before re-rating the item and can therefore change their initial responses based on the feedback from the previous rounds. Previous research outside the context of core sets has demonstrated that both the iteration of questionnaires, enabling participants to reflect on their own previous responses, and the influence of feedback, improve accuracy of responses and agreement amongst participants [8, 9]. Whilst accuracy cannot be assessed in the context of a core set, since there is no ‘correct’ result, ensuring some degree of consensus is paramount.

Research in social psychology has suggested that different presentations of feedback will lead to differences in change of opinion between rounds [9, 10], however evidence-based guidelines on how best to provide this feedback do not exist [11–13]. Whilst most Delphi present feedback in the form of summary statistics [11], the majority of research has focussed on the impact of the presentation of rationale in addition to summary statistics [12], rather than comparing different presentations of quantitative data.

In the context of core sets and elsewhere, the selection of participants or stakeholders in a Delphi is crucial to ensure diversity in views [1, 13, 14]. With no communication between participants the presentation of feedback is the only mechanism for reconciling different opinions of participants. However, the responses from such a heterogeneous group of participants are generally fed back as an overall average [12, 13], which will be heavily dependent on the participant mix and will conceal any disparate views between stakeholders. The process could, alternatively, be performed for each stakeholder group separately, presenting feedback from a participant’s own stakeholder group only and differences in items prioritised using these two methods have been observed [15].

A better approach may be to feedback to all participants the average responses of each stakeholder group separately such that items with no consensus can be deliberated further. In a recent study, by Harman and colleagues, health professionals completed multiple rounds which included feedback from different stakeholder groups in different rounds [16]. The results suggested that the responses of parents and children and other health professional groups had a different impact on the perceived importance of outcomes compared to those of their peer group alone. This now needs to be evaluated in a randomized study.

This study presents exploratory work to consider the following hypotheses, in the context of Delphi studies for core set development:

1. There is a difference between peer group only and multiple group feedback in terms of subsequent responses and the magnitude of change
2. There is a difference between peer group only and multiple group feedback in terms of items retained at the end of a Delphi study
3. There is a difference between peer group only and multiple group feedback in terms of the level of agreement between stakeholder groups

## Methods

This methodological work employed three parallel randomized controlled trials, nested within the development of three core sets: a COS for surgery for colorectal cancer [17]; a COS for breast reconstruction surgery [18]; and a CIS for surgery for oesophageal cancer [3]. For all three studies, Delphi questionnaires were developed after identification of a long list of all possible outcomes from a literature review and interviews with patients [19–23]. The long list was mapped into outcome/information domains, which were included as individual items in a round 1 questionnaire to use in the Delphi study. Items were written in lay terms with medical terms in brackets so that they could be understood by all. Participants were asked to rate the importance of each item from 1 (not essential) to 9 (absolutely essential). For each of the core sets the Delphi process consisted of two rounds of questionnaires, completed by patients and health professionals.

**Fig. 1:**
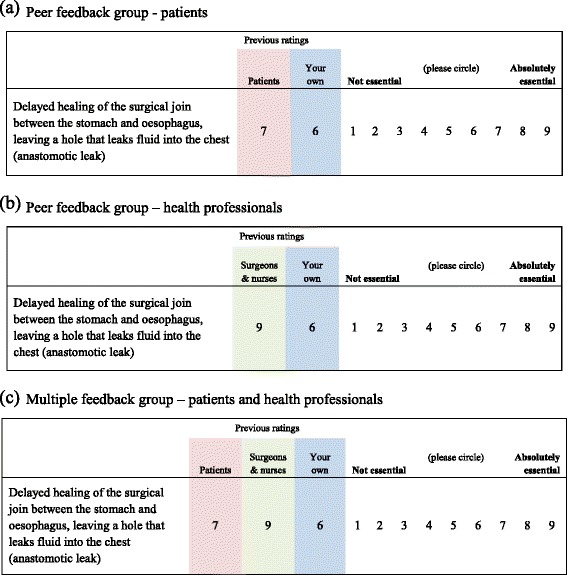
Feedback presented in round 2 questionnaires, example from oesophageal core information set (CIS). Participants were asked to "Please complete this questionnaire and circle the score that best represents your opinion regarding whether each topic should be discussed with patients prior to surgery."Previous ratings are shown here as mean scores from round 1

Within each study, patients and professionals completing round 1 received a second questionnaire (round 2) which included, for each item, the individual’s own score from round 1 and group feedback from round 1 (Fig. 1). For the group feedback, participants (who were not deceased) were randomized, using a computer-generated schedule (developed by the study statistician), to receive summary data from their own stakeholder group only (‘peer’ feedback) or from both patients and health professionals separately (‘multiple’ feedback) in a 1:1 ratio. Randomization for each of the three studies was stratified by stakeholder group. The allocation schedule was used (within a mail-merge) to automatically generate the allocated questionnaire for each participant. For the colorectal and oesophageal studies feedback from round 1 consisted of mean scores, chosen for simplicity. The mean scores for each item were calculated for all patients completing the round 1 questionnaire and all health professionals completing round 1 separately. For the breast reconstruction study (which was the last to occur) the percentage scoring between 7 and 9 was used, which was felt to better demonstrate differences between the stakeholder groups than mean scores. Participants were asked to consider the feedback and re-rate the items. Decisions as to which items should be retained following each round were determined by pre-specified statistical criteria (see below). Items retained at the end of round 2 were considered further in subsequent face-to-face consensus meetings and a final core set agreed; these are reported elsewhere [3, 17, 18].

### Statistical analyses

In order to address the study hypotheses, analyses considered: differences between peer group and multiple group feedback in terms of (a) subsequent scores and the magnitude of opinion change (hypothesis 1); (b) items retained at the end of the Delphi (hypothesis 2); and (c) the level of agreement between stakeholders (hypothesis 3). Since analyses were conducted in three separate datasets, relating to different clinical scenarios, this also afforded some opportunity to explore whether the effects observed differed depending on the initial level of agreement between stakeholders.

#### Sample size

The nested trials were based on opportunistic samples participating in the Delphi processes of core set development, rather than any pre-determined calculation for this methodological work. As a consequence statistical testing is largely exploratory and formative.

#### Criteria for retaining items

At the end of round 1, items were retained for round 2 if they were scored between 7 and 9 by 50 % or over of respondents and between 1 and 3 by less than 15 %. These criteria were considered separately for patient and health professional groups. For the oesophageal core information set (which was the first set developed by the team), different versions of the round 2 questionnaire were created depending on the intended stakeholder group. The round 2 questionnaire for patients randomized to peer feedback included only items retained by patients in round 1; the questionnaire for professionals randomized to peer feedback included items retained by professionals in round 1; and all participants randomized to multiple feedback received a questionnaire including all items retained by either group. It subsequently became apparent that for the purposes of this methodological work it would have been advantageous for all participants to have received the same round 2 questionnaire. Hence, for the colorectal and breast reconstruction core sets all participants received a round 2 questionnaire including all items retained by patients or professionals.

Items to retain at the end of round 2 were considered with stricter cut-off criteria; retaining items scored between 7 and 9 by 70 % or over and between 1 and 3 by less than 15 %. Retained items were identified for patients and professionals separately.

#### Initial agreement between stakeholders

Initial agreement between patients and professionals in round 1 scores was assessed in two ways. First, for each item in turn, the percentage scoring 1 to 3, 4 to 6 and 7 to 9 was calculated for patients and professionals separately. Items were ranked depending on the percentage of participants scoring the item between 7 and 9 (defined as ‘essential’) and the ‘top 10’ items compared between stakeholder groups. Second, items to be retained for round 2 (using the pre-specified criteria) were identified, and the percentage of items for which there was agreement between stakeholder groups was calculated (that is, the percentage of items retained by neither stakeholder group, or both stakeholder groups).

#### Differences between peer group and multiple group feedback in terms of subsequent scores and the magnitude of opinion change (hypothesis 1)

The percentage of items for which a participant changed their score between rounds 1 and 2 was calculated, along with the mean absolute change in score (ignoring the direction of change) for each participant. These were then compared between randomization groups using independent *t* tests. Analyses were considered separately for patients and professionals. In addition, for each item, a linear regression compared round 2 scores between feedback groups, adjusting for round 1 scores. Given the number of statistical tests performed 5 % of tests were expected to result in a *P* value ≤ 0.05 by chance; we therefore examined the percentage of tests with *P* ≤ 0.05 in relation to this expected percentage.

#### Differences between peer group and multiple group feedback in terms of items retained at the end of the Delphi (hypothesis 2)

For each stakeholder group two-by-two contingency tables categorised the number of items retained at the end of round 2 by (i) both the peer and multiple feedback groups; (ii) the peer feedback group only; (iii) the multiple feedback group only; and (iv) neither feedback group. The percentage of items for which there was agreement between the feedback groups was calculated along with the percentage of discordant items, retained by one feedback group but not the other. This was performed separately for patients and professionals.

#### Differences between peer group and multiple group feedback in terms of the level of agreement between stakeholders (hypothesis 3)

For each feedback group two-by-two contingency tables categorised the number of items retained by (i) both stakeholder groups; (ii) patients only; (iii) health professionals only; and (iv) neither stakeholder group. The percentage agreement and percentage of discordant items were calculated.

To further explore the impact of feedback on subsequent consensus between stakeholders the following analyses were employed. Amongst participants randomized to peer feedback only, for each item, the absolute difference (ignoring direction) in mean patient round 2 score and mean professional round 2 score was obtained using linear regression. The regression adjusted also for participants’ round 1 score for that respective item. This was then repeated amongst participants randomized to the multiple feedback group and again the absolute difference in means between stakeholders generated for each item. The unit of analysis then became questionnaire item rather than participant, with each observation an aggregate summary statistic. Absolute mean differences (between stakeholders) across the questionnaire items were compared between the peer and multiple feedback groups using a paired *t* test.

Finally, responses of patients and professionals were amalgamated within each of the randomization arms (that is, completely ignoring stakeholder group) and the standard deviation calculated for each item, as a measure of the spread in responses across stakeholders, for each feedback group. This was done separately for round 1 and round 2 and the reduction in each item’s variability between rounds calculated. Again, the unit of analysis became item with each observation an aggregate summary statistic. The mean reductions in standard deviation were then compared, across all items, between the peer and multiple feedback arms using a paired *t* test.

For all statistical tests 95 % confidence intervals and *P* values were derived and all analyses were performed in Stata version 13 [24].

## Results

The round 1 questionnaire contained 93 items for the colorectal cancer study, 34 for breast reconstruction and 67 for oesophageal cancer. Numbers invited to participate for each core set, and the percentage of participants completing questionnaires are presented in Table 1. Initial response rates for round 1 varied between stakeholder groups and core sets. Response rates for round 2 were high for both stakeholder groups across all three core sets (in excess of 74 % for all) (Table 1). All participants were recruited from the UK with the exception of the oesophageal set in which approximately 50 % of patients and 20 % of health professionals were recruited from the Netherlands. The types of health professionals included differed across the core sets as appropriate but all included surgeons and nurse specialists. Full details of participant demographics are provided in the relevant core outcome set articles [3, 17, 18].

**Table 1:** Numbers (%) completing round 1 and round 2 questionnaires

Core set	Round 1		Round 2	
	Patients	Health professionals	Patients	Health professionals
Colorectal	97/267 (36.3 %)	98/321 (30.5 %)	87^a^ /97 (89.7 %)	78/98 (79.6 %)
Breast	215/434 (49.5 %)	88/156 (56.4 %)	190/214 (88.8 %)	69/88 (78.4 %)
Oesophageal	185/286 (64.7 %)	126/230 (54.8 %)	145/166^b^ (84.5 %)	107/126 (84.9 %)

^a^Whilst 45 patients randomized to the multiple feedback group returned a questionnaire, one patient only completed items related to other aspects of the research not reported here; all round 2 core set outcomes were missing

^b^Eleven patients completing round 1 died and eight were too ill to complete round 2

### Initial agreement between stakeholders

Within the colorectal study there was a low level of initial agreement between stakeholders, with only five overlapping items that both stakeholder groups ranked in their top 10 for measurement in a trial (see Additional file 1: Table S1). According to criteria specified *a priori* (items rated 7–9 by 50 % or over and 1–3 by less than 15 %) 47 of the 93 items were retained by neither group and 15 by both stakeholder groups, hence there was 67 % agreement between stakeholder groups; the remaining 31 items were retained by one stakeholder group but dropped by the other. A total of 46 items of the original 93 were taken forward into the round 2 questionnaire (items retained by either patients, professionals or both).

In the breast reconstruction study there was far more agreement between the stakeholder groups, with nine items common to both the patients’ and health professionals’ top 10 (see Additional file 1: Table S1). In this instance there was 91 % agreement (31 items retained by both stakeholder groups); the remaining three items were retained by one group but not the other, hence all 34 items were retained for round 2.

Consensus between stakeholders was again low within the oesophageal study, with only four items appearing in both the patients’ and health professionals’ top 10 most essential items to be disclosed in a consultation (see Additional file 1: Table S1). As with the colorectal study, there was 67 % agreement in items retained (29 retained by both groups, 16 by neither), and a total of 51 items retained for round 2 (51 items in multiple feedback group questionnaires, 44 in patient peer group, 36 in professional peer group questionnaires (see Methods)).

### Baseline comparison of randomization groups

**Fig. 2:**
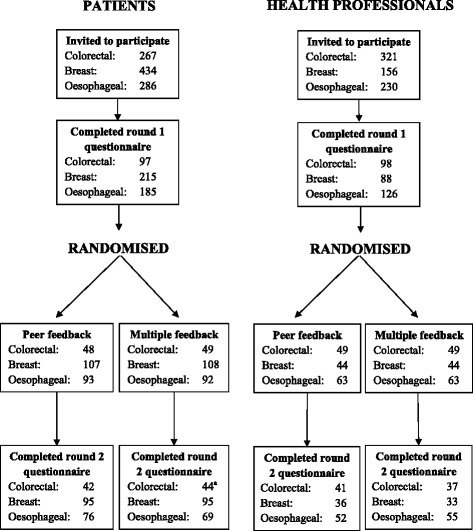
Flow diagram for colorectal cancer surgery, breast reconstruction and oesophageal cancer surgery core sets. ^a^Whilst 45 patients randomized to the dual feedback group returned a questionnaire, one patient only completed items related to other aspects of the research not reported here; all round 2 scores were missing

For each core set, all those completing round 1 were sent a round 2 questionnaire (with the exception of 19 patients in the oesophageal study who had died or were too ill to participate) including items retained from round 1. The numbers randomized to receive peer feedback only (from their own stakeholder group) and multiple feedback (from both stakeholder groups separately) are presented in Fig. 2. Clinical and socio-demographic details of patients and speciality of health professionals were similar between the randomized arms for all three studies (Table 2). Round 1 item scores were also similar; differences in mean (and median) scores were less than 1 for 89–100 % of items across the three studies and no more than 2 for all items. As expected given the categorisation of data, some larger differences were seen between the peer and multiple feedback groups in terms of the percentage rating an item 7–9 (there was less than a 10 % difference between feedback groups for 71–91 % of items across the three sets; and less than a 15 % difference for between 86 % and 99 % of items). Discrepancies between the randomization groups were greatest amongst the core sets and stakeholder groups with the fewest numbers.

**Table 2:** Baseline demographics and job speciality for participants completing round 2

	Colorectal COS		Breast reconstruction COS		Oesophageal CIS	
	Peer feedback	Multiple feedback	Peer feedback	Multiple feedback	Peer feedback	Multiple feedback
Patients	*N* = 42	*N* = 44	*N* = 95	*N* = 95	*N* = 76	*N* = 69
Male (%)	21 (50)	27 (61)	0	0	57 (75)	52 (75)
Mean age (SD)	63.2 (11)	68.6 (10)	54.4 (8)	54.6 (9)	66.9 (7)	66.2 (9)
Education above GSCE (%)	24 (57)	28 (64)	60 (63)	65 (68)	39 (51)	43 (62)
Married/co-habiting (%)	37 (88)	35 (80)	69 (73)	69 (73)	60 (79)	55 (79)
Working full/part-time (%)	11 (26)	11 (25)	62 (65)	56 (59)	17 (22)	9 (13)
Mean time since surgery (years)	4.4	3.2	2.3	1.8	2.5	3.2
Second operation needed (%)	20 (48)	24 (55)	nr	nr	12 (16)	12 (17)
Hospital stay >2 weeks (%)	6 (14)	8 (18)	nr	nr	30 (39)	22 (32)
Health professionals	*N = 41*	*N = 37*	*N = 36*	*N = 33*	*N = 52*	*N = 55*
Male (%)	28 (74)	28 (76)	17 (47)	15 (45)	40 (77)	39 (71)
Age 40 or more (%)	35 (92)	33 (89)	36 (100)	30 (91)	40 (77)	39 (71)
Consultant surgeon (%)	35 (92)	32 (86)	28 (78)	22 (67)	38 (73)	37 (67)
Clinical specialist nurse (%)	2 (5)	4 (11)	7 (19)	8 (24)	9 (17)	13 (24)

*COS* core outcome set, *CIS* core information set, *nr* not recorded

#### Differences between peer group and multiple group feedback in terms of subsequent scores and the magnitude of opinion change (hypothesis 1)

**Fig. 3:**
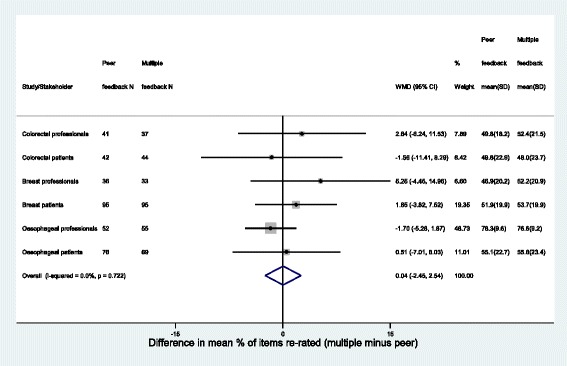
Forest plot of difference in percentage of items re-rated between peer group and multiple group feedback. WMD Weighted mean Difference relates to overall estimate only; I-Squared demonstrates little heterogeneity, fixed effects model presented

**Fig. 4:**
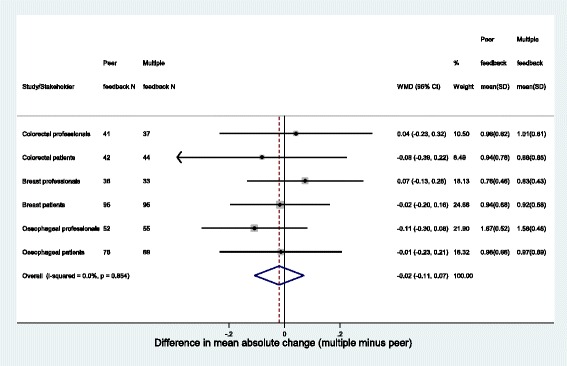
Forest plot of difference in mean absolute change between rounds between peer group and multiple group feedback. WMD Weighted Mean Difference relates to overall estimate only; I-Squared demonstrates little heterogeneity, fixed effects model presented

There was very little difference seen between the peer and multiple feedback groups for either patients or professionals in terms of the percentage of items for which a participant changed their score (re-rated) between rounds 1 and 2. This was true for each of the core sets (Fig. 3). Participants re-rated approximately 50 % of items irrespective of feedback group, stakeholder group or core set. The only exception to this was amongst professionals in the oesophageal cancer study who re-rated over 75 % of items, irrespective of what feedback they received from round 1. Similarly, there was little difference in the absolute change in scores between the feedback groups amongst patients or health professionals (Fig. 4). The mean absolute change in scores (ignoring the direction of change) varied between 0.76 points and 1.67 points, with the highest values again seen amongst the oesophageal cancer professionals.

Differences were observed between the randomization groups however, in terms of actual round 2 scores (adjusting for round 1). Within the breast reconstruction study, for which initial consensus between stakeholders had been high, only one item (out of 34) demonstrated any evidence of a difference between the peer and multiple patient feedback groups, and one item between the peer and multiple health professional groups. However, amongst the two cancer studies where initial consensus between stakeholders was much lower, a higher number of items demonstrated evidence of a difference between the feedback groups: four of 46 items (8.7 %) and five of 46 items (10.9 %) amongst colorectal patients and professionals respectively; three of 44 items (6.8 %) and seven of 36 items (19.4 %) amongst oesophageal patients and professionals respectively.

#### Differences between peer group and multiple group feedback in terms of items retained at the end of the Delphi (hypothesis 2)

Applying the pre-specified criteria for retaining items at the end of round 2, each core set contained discordant items, where an item was retained by one feedback group and not the other (Table 3). The degree of discrepancy was dependent on the core set and stakeholder group but varied between 3 % and 22 % of items, with the highest degree of discordant items amongst the colorectal patients and the oesophageal health professionals.

**Table 3:** Number of items retained at end of round 2 by peer and multiple feedback groups

Core set/Stakeholder group	Number of participants		Items retained at end of round 2^a^, no. (%)				% discordant items	% agreement
	Peer feedback group	Multiple feedback group	Retained by both feedback groups	Retained by peer feedback group only	Retained by multiple feedback group only	Retained by neither feedback group		
Colorectal								
Patients	42	44	9/46	6/46	4/46	27/46	22 %	78 %
Professionals	41	37	19/46	2/46	0/46	25/46	4 %	96 %
Breast								
Patients	95	95	19/34	1/34	0/34	14/34	3 %	97 %
Professionals	36	33	16/34	2/34	1/34	15/34	9 %	91 %
Oesophageal								
Patients	76	69	18/44	2/44	0/44	24/44	5 %	95 %
Professionals	52	55	15/36	1/36	6/36	14/36	19 %	81 %

^a^Items were retained by each feedback group if they were scored between 7 and 9 by 70 % or more and between 1 and 3 by less than 15 %

#### Differences between peer group and multiple group feedback in terms of the level of agreement between stakeholders (hypothesis 2)

In terms of the items retained at the end of round 2, there were consistently fewer discordant items and greater agreement between patients and professionals amongst those randomized to receive multiple group feedback compared to peer feedback only, within all three core set studies. Indeed, similar absolute improvements in agreement were seen in the three studies (9 %, 12 % and 13 % for the colorectal, breast and oesophageal studies respectively) (Table 4).

**Table 4:** Number of items retained at end of round 2 by patients and health professionals

Core set/Feedback group	Number of participants		Items retained at end of round 2^a^, no. (%)				% discordant items	% agreement
	Patients	Professionals	Retained by both stakeholder groups	Retained by patients only	Retained by health professionals only	Retained by neither stakeholder group		
Colorectal								
Peer feedback	42	41	10/46	5/46	11/46	20/46	35 %	65 %
Multiple feedback	44	37	10/46	3/46	9/46	24/46	26 %	74 %
Breast								
Peer feedback	95	36	16/34	4/34	2/34	12/34	18 %	82 %
Multiple feedback	95	33	17/34	2/34	0/34	15/34	6 %	94 %
Oesophageal								
Peer feedback	76	52	11/29	6/29	4/29	8/29	35 %	65 %
Multiple feedback	69	55	14/51	4/51	7/51	26/51	22 %	78 %

^a^Items were retained by each stakeholder group if they were scored between 7 and 9 by 70 % or more and between 1 and 3 by less than 15 %

Absolute differences in mean round 2 item scores (adjusting for round 1) were smaller between patients and professionals receiving multiple group feedback than between those receiving their peer group feedback only for all three studies (Table 5). In addition, for all three studies there was strong evidence of reduced variability in round 2 scores amongst stakeholders receiving multiple compared to peer feedback. However, the actual magnitude of this reduction was relatively small (Table 6).

**Table 5:** Comparison of differences between stakeholders between peer and multiple feedback groups

Core set	Mean absolute difference in mean round 2 scores between patients and professionals^a^ (SD)		Difference in means (peer-multiple) (95 % CI); *P* value^c^
	Peer feedback	Multiple feedback	
Colorectal	0.54 (0.40)	0.42 (0.30)	0.12 (−0.02 to 0.26); *P* = 0.081
Breast	0.31 (0.21)	0.14 (0.14)	0.17 (0.08 to 0.27); *P* < 0.001
Oesophageal^b^	0.40 (0.30)	0.23 (0.17)	0.17 (0.09 to 0.26); *P* < 0.001

^a^Linear regression adjusting for round 1 scores employed to generate absolute differences

^b^Based on the 29 items included in all round 2 questionnaires

^c^ *P* value from paired *t* test

**Table 6:** Variability in rounds 1 and 2 scores combining stakeholder groups – comparison of peer and multiple feedback groups

Core set	Mean SD (SD)						Difference in mean reduction (peer minus multiple)^a^
	Peer			Multiple			
	Round 1	Round 2	Mean reduction	Round 1	Round 2	Mean reduction	
Colorectal	2.07 (0.31)	1.73 (0.34)	0.34 (0.21)	2.25 (0.35)	1.73 (0.28)	0.51 (0.16)	−0.18 (−0.26 to −0.09); *P* < 0.001
Breast	1.62 (0.36)	1.66 (0.31)	−0.04 (0.15)	1.59 (0.38)	1.54 (0.28)	0.05 (0.17)	−0.08 (−0.14 to −0.03); *P* = 0.005
Oesophageal^b^	1.88 (0.22)	1.69 (0.17)	0.19 (0.18)	1.81 (0.24)	1.37 (0.19)	0.45 (0.11)	−0.26 (−0.35 to −0.17); *P* < 0.0001

^a^ *P* value from paired *t* test

^b^Based on the 29 items included in all round 2 questionnaires

## Discussion

This methodological work examined the impact of providing feedback on different stakeholders groups’ opinion in Delphi methodology to gain consensus as to what constitutes core outcome and core information sets in three clinical areas. Providing feedback on both patient and professional opinion (multiple feedback group), rather than a participant’s peer stakeholder group only, did not lead to any more or less items being re-rated in round 2 (Fig. 3). Neither was there an impact on the average amount by which item scores were changed (Fig. 3). There was some evidence of an impact however on actual item scores in round 2 in the two cancer surgery studies, suggesting that the direction of change in scores between rounds was related to feedback received. The differences observed did not always lead to disparity in whether items were subsequently retained (for example, where the percentage scoring 7–9 remained < 70 % in both feedback groups irrespective of differences in mean actual scores).

Items retained at the end of the Delphi process were however influenced by feedback group (Table 3). The extent of this was dependent on the initial level of agreement between stakeholder groups; with a greater impact amongst studies where initial agreement was poorer. Within the two cancer studies the impact was also dependent on stakeholder group; for example, type of feedback had a bigger impact amongst colorectal patients than professionals.

Also explored within this current work was whether providing feedback from both stakeholder groups improved overall agreement. Following feedback, differences in item scores and variability in scores between patients and professionals were smaller amongst those receiving feedback from both stakeholder groups rather than their peer group only (Tables 5 and 6). Again, of more importance was that agreement between patients and professionals in terms of the items retained was greatest amongst those receiving feedback from both stakeholder groups for all three studies (Table 4).

These findings agree with and extend previous non-randomized methodological work [16]. We are only aware of one previous study that randomized participants to receive feedback from different stakeholder groups within a Delphi process [15]. Campbell and colleagues randomized physicians and health care managers, assessing quality indicators for primary care, to receive peer group only or whole group feedback (as opposed to both groups separately). They also observed differences between feedback groups in terms of items considered as valid measures.

In the present study, the reasons for discrepancies in items retained between the feedback groups were not always clear. For approximately a third of the discrepant items across each core set, differences between the feedback groups were minor (less than 5 % difference in percentage rating an item 7–9). Some reasons for larger discrepancies were more transparent than others. For example, in round 1 of the colorectal set, patients rated passing blood higher than professionals (mean scores of 7 versus 5). In round 2, patients, seeing professional as well as patient feedback (multiple feedback) were less likely to rate it as essential than those receiving patient feedback only (55 % compared to 79 %); patients potentially reducing their initial scores in line with health professionals’ views. The reasons for other discrepancies were less clear: for example, within the colorectal set, lymph node harvest was rated lower in round 1 by patients than professionals (mean score 7 versus 8), so one might expect patients receiving professionals’ feedback to rate the item more highly in round 2. However, in round 2 only 56 % of the patient multiple feedback group rated it as essential compared to 74 % of patients in the peer feedback group. There were also examples where patient and professional feedback were identical, but subsequent discrepancies were seen between the randomization groups in round 2 scores; and examples where large differences in the round 1 feedback resulted in very little difference between the randomization groups in round 2 scores. These findings agree with work by Campbell and colleagues who conducted interviews with stakeholders involved in a Delphi exercise to develop a set of quality indicators for the organisation and delivery of primary mental health care. They found that the processes involved in interpreting a question and formulating an answer were complex and that participants often had different reasons for giving the same response [25]. Bardecki examined the psychological structures involved in opinion change within the Delphi and suggested that the degree of cognitive dissonance (where a participant is confronted with new information that conflicts their existing beliefs) played an important role in judgements being shifted towards the summary statistic [26]. Rowe and Wright conceptualized change in opinion as resulting from both internal Delphi process factors, such as the degree of expertise and confidence of a participant [9, 27], and external factors such as the nature of the feedback and whether the task is ‘intellective’ or ‘judgmental’.

We did not collect data on participants’ understanding of core outcome sets (or for example whether professionals were also academics with familiarity in outcomes research) and differences in knowledge may lead to different prioritisation of items. Previous research has suggested that higher expertise is associated with less change in opinion between rounds and that the extent of this may depend on the nature of feedback [8]. Degree of change may also be associated with level of confidence in a participant’s judgements [27]. In the present study, due to the randomization, knowledge and confidence are likely to be balanced between the randomized groups and so are unlikely to impact on our findings. This is, however, an area for further research.

For the two cancer studies, feedback was presented as a mean value (integer) in order to be easily understood. For the breast reconstruction set the percentage rating an item 7–9 was presented as it better demonstrated discordant views between stakeholders. The actual summary statistics used may also impact on opinion change – Bardecki describes the credibility of the summary measure (the ‘communicator’ or ‘anchor’) and suggests that a perfectly credible anchor would be more likely to induce ‘assimilation’, that is a shift in judgement towards the anchor [26]. Hence opinion change may also depend on the respondents’ perception of the credibility of the summary measure.

It has been suggested that rationale for responses should also be fed back to participants [12, 28], or that only rationale should be presented to prevent participants simply conforming with the majority [27]. Indeed, as described earlier, the majority of feedback research has focussed on the inclusion of rationale. Meijering and colleagues randomized participants to receive rationale only or rationale plus summary statistics and found no impact on the degree of change in opinion, but interestingly less agreement amongst those receiving rationale only [12]. We are not aware of any research comparing summary statistics with summary statistics plus rationale and it would be interesting to see if the addition of rationale impacted on subsequent rescoring.

In each of the three studies in this work there was a degree of attrition between rounds (ranging from 11 % to 26 %) (Table 1). Previous research suggests that those with minority opinions are more likely to drop out [8]. In this study, participant beliefs are likely to have been balanced between the randomized feedback groups (indeed there were few differences between the randomized groups in terms of round 1 scores) and the rate of dropout was similar within the groups (Fig. 2). However, there is still some potential for attrition bias and this could be investigated further.

We chose cut-off criteria for retaining items after discussions with other academics developing core outcome sets and members of the COMET Initiative; but the definition of what constitutes consensus varies widely across studies [29]. Items retained after each round of a Delphi exercise are entirely dependent on the definition of consensus used, so consideration is needed of how different criteria might have impacted on the results. Since differences were observed in actual item scores following receipt of the peer-only and multiple feedback, there would most likely also be disagreement between the feedback groups in terms of which items should be retained, whatever consensus criteria was used.

Consideration should also be given to the participants included in the three studies. Patients and health professionals were the only stakeholders recruited as these were considered the key groups to inform the core sets [13]. Delphi participants need to have relevant expertise in the condition or treatment to be able to prioritise items and other stakeholders such as methodologists, regulators and industry representatives may be unable to carry out this task (although they may add value to other stages of the development of a core set, such as the decision of how to measure an outcome or the implementation of a core set). The majority of participants in the three studies were from the UK, again it is plausible that in different countries patients and health professionals may react differently to feedback from each other’s stakeholder groups, although we are unaware of any empirical evidence to support this. At present, this study provides the best evidence on which to base recommendations, but should be repeated in other settings and countries.

Feedback is a key characteristic of the Delphi process; understanding how participants perceive and use this feedback is paramount to the future optimal design of such methodology. Future qualitative work might further improve our understanding of the underlying mechanisms influencing opinion change between Delphi rounds in the context of core outcome or information sets. For example, ‘Think aloud’ cognitive interviews [30], conducted whilst participants complete a Delphi questionnaire, might focus on how a respondent makes the decision to initially score an item and how responses are subsequently influenced by feedback from different stakeholder groups. This would further inform the most appropriate methods to be used in the future.

## Conclusions

In the development of a core outcome or information set, the level of agreement between stakeholder groups depends on the feedback presented, even when initial agreement between stakeholders is high. Type of feedback will also impact on the items subsequently retained at the end of a Delphi process (used to inform subsequent consensus meetings and the final core set). We would recommend providing all participants with feedback from each stakeholder group separately, since this may improve agreement between stakeholder groups by enabling reflection on other groups’ views. Further work is needed to better understand this process.

## Abbreviations

CIS, core information set; COS, core outcome set; REC, Research Ethics Committee

## Additional file

Additional file 1: Table S1. Top 10 items rated essential in round 1. (DOCX 17 kb)
