# Supplementary material for: Three nested randomized controlled trials of peer-only or multiple stakeholder group feedback within Delphi surveys during core outcome and information set development
Source: Trials. 2016 Aug 17;17:409. doi: 10.1186/s13063-016-1479-x (PMC4989325; doi:10.1186/s13063-016-1479-x)
Supplement: Additional file 1: Table S1. — Top 10 items rated essential in round 1. (DOCX 17 kb) [file 13063_2016_1479_MOESM1_ESM.docx]

**Additional file 1: Table S1. Top 10 items rated essential in round 1**

| **Core Set** | **Health professionals** | | **Patients** | |
| --- | --- | --- | --- | --- |
|  | **Item** | **% rating 7-9** | **Item** | **% rating 7-9** |
| **Colorectal COS** | Anastomotic leak | 99.0 | *Resection margins* | 90.6 |
|  | *Resection margins* | 95.8 | *Stoma rate* | 87.4 |
|  | Operative mortality | 91.7 | *Distant recurrence* | 83.2 |
|  | Conversion to open operation | 90.6 | *Recurrence* | 81.9 |
|  | *Distant recurrence* | 89.6 | *Local recurrence* | 81.9 |
|  | Reoperation | 89.6 | Non-progression | 81.5 |
|  | *Local recurrence* | 89.5 | Disease-free interval | 80.9 |
|  | *Recurrence* | 87.5 | Sphincter preservation | 75.5 |
|  | *Stoma rate* | 87.4 | *Lymph node yield* | 73.7 |
|  | *Lymph node yield* | 86.5 | Survival | 72.9 |
|  | Length of hospital stay | 86.5 | Physical function | 72.9 |
|  | Unplanned readmission | 86.5 |  |  |
| **Breast COS** | *Quality of life* | 97.7 | *Women’s cosmetic satisfaction* | 92.6 |
|  | *Women’s cosmetic satisfaction* | 96.6 | *Major complications* | 92.1 |
|  | *Patient reported cosmetic outcome* | 93.2 | *Patient reported cosmetic outcome* | 91.6 |
|  | *Implant related complications* | 92.1 | *Flap related complications* | 89.7 |
|  | *Flap related complications* | 92.1 | *Implant related complications* | 87.7 |
|  | *Major complications* | 89.9 | *Quality of life* | 87.4 |
|  | *Body image* | 89.8 | *Normality* | 86.1 |
|  | Self-esteem | 87.5 | Wound related complications | 85.1 |
|  | *Emotional well-being* | 87.5 | *Body image* | 84.7 |
|  | *Normality* | 86.2 | *Emotional well-being* | 83.7 |
|  |  |  | Unplanned surgery | 83.7 |
| **Oesophageal CIS** | Anastomotic leak | 97.6 | Cancer recurrence | 86.0 |
|  | In-hospital death | 92.1 | Survival | 84.9 |
|  | Type of surgery | 91.3 | *Long term quality of life* | 84.6 |
|  | In-hospital recovery | 88.1 | Long term general health | 83.2 |
|  | *Long term quality of life* | 88.0 | *Information about risks related to comorbidites* | 82.6 |
|  | Respiratory complications | 87.3 | *Inoperability* | 82.2 |
|  | *Recovery after discharge* | 87.3 | Physical function | 81.0 |
|  | *Information about risks related to comorbidities* | 86.5 | *Recovery after discharge* | 80.4 |
|  | *Inoperability* | 85.7 | Anastomotic stricture | 80.0 |
|  | Re-operation | 84.9 | Follow-up arrangements | 79.9 |

Items in italics were prioritised in the top 10 items for both patients’ and professionals’
